# A Case Report and Literature Review of an Uncommon Manifestation of Giardiasis: Protein-Losing Enteropathy in an Immunocompetent Host

**DOI:** 10.7759/cureus.82601

**Published:** 2025-04-19

**Authors:** Shubhransu Patro, Vibha Sharma, Dipleshdeep Goyal, Gyanamitra Panigrahi, Sidharth S Pattnaik

**Affiliations:** 1 Internal Medicine, Kalinga Institute of Medical Sciences, Bhubaneswar, IND

**Keywords:** diarrhea, giardiasis, hypoproteinemia, malnourished, metronidazole, parasite, protein-losing enteropathy, public health, tinidazole

## Abstract

Hypoproteinemia can result from multiple etiologies, including malnutrition, hepatic and renal dysfunction, and less commonly, protein-losing enteropathy (PLE). PLE leads to protein loss into the gastrointestinal tract, resulting in decreased oncotic pressure and third-space fluid accumulation. While enteric infections have been implicated in PLE, *Giardia lamblia* is a rare causative agent, particularly in immunocompetent adults. We discuss the case of a 41-year-old immunocompetent male who presented with progressive generalized edema, weight gain, and skin hypopigmentation over the past six months. Laboratory investigations revealed significant hypoalbuminemia (1.6 g/dL). An extensive evaluation ruled out hepatic, renal, and gastrointestinal pathologies, but the stool analysis confirmed *Giardia lamblia *cysts, with a positive *Giardia *antigen test. The patient was treated with a single dose of tinidazole (2 g) and intravenous albumin. Follow-up after two months showed resolution of edema, weight loss, and improved serum albumin levels (2.7 g/dL) and negative stool cultures. While giardiasis typically causes diarrheal illness, this case highlights its rare but significant role in PLE, even in immunocompetent adults. Clinicians should consider stool testing for *Giardia* in unexplained hypoalbuminemia, as prompt treatment can reverse protein loss and prevent complications. The pathophysiology involves mucosal damage and increased intestinal permeability, leading to excessive protein loss. The few cases of giardiasis-associated PLE highlight the need for heightened clinical suspicion as early recognition and prompt treatment with tinidazole can lead to favorable outcomes, preventing complications associated with prolonged infection.

## Introduction

Patients presenting with hypoproteinemia have various underlying etiologies, including malnutrition, hepatic and renal dysfunction, and less commonly, protein-losing enteropathy (PLE). PLE is rare in giardiasis, with most cases reported in children or immunocompromised hosts. This report underscores its occurrence in immunocompetent adults, emphasizing the need for stool testing in unexplained hypoalbuminemia, even without classic diarrhea. It manifests clinically as ascites, pleural effusion, and pedal edema, and can contribute to malnutrition over time [1]. The primary causes of gastrointestinal protein loss fall into three main categories: primary erosive gastrointestinal disorders, non-erosive gastrointestinal disorders, and conditions leading to increased interstitial pressure or lymphatic obstruction [2]. Enteric infections caused by pathogens such as *Salmonella, Shigella, Clostridium difficile*, viruses, and parasites can compromise epithelial integrity, leading to protein loss, particularly in immunocompromised patients [3].

Giardiasis, caused by the protozoan *Giardia intestinalis *(also known as *Giardia lamblia* or *Giardia duodenalis*), is one such enteric infection and remains a globally prevalent parasitic disease, affecting an estimated 200 million people annually [4]. It is a major cause of diarrheal illness along with Cryptosporidium worldwide, particularly among children under five [5]. In industrialized nations, giardiasis is often linked to waterborne outbreaks, particularly in recreational water settings such as lakes and swimming pools [6]. Risk factors for infection include poor sanitation, contaminated drinking water, and zoonotic transmission from domestic animals such as dogs and cats [4]. While the prognosis for patients with giardiasis is generally good as most infections are self limiting, mortality risks are higher with infants and malnourished children [7].

## Case presentation

**Figure 1:**
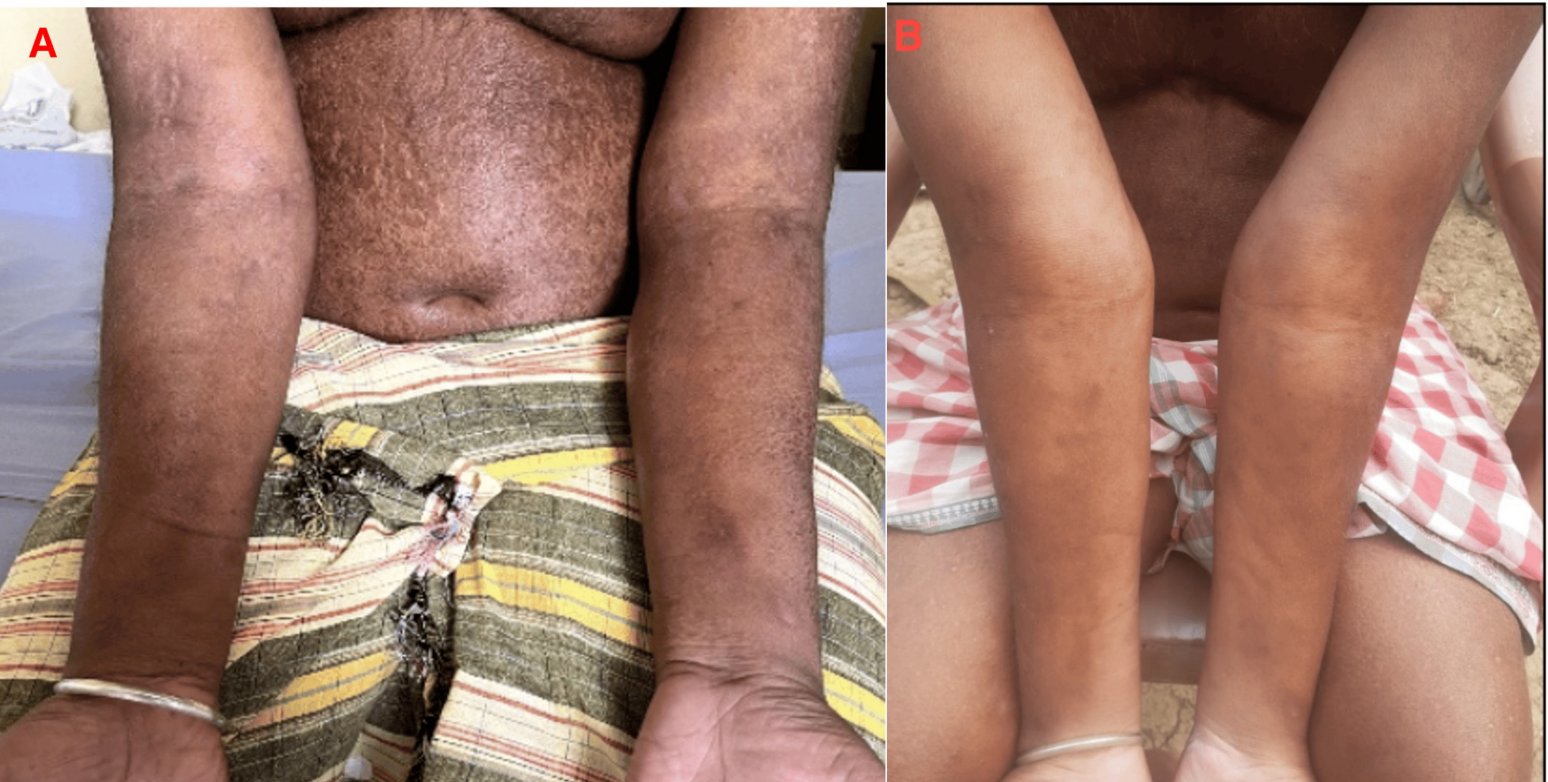
Upper extremities of the patient A: Edema of the upper extremities and skin changes on presentation B: Resolution of the edema and skin changes after treatment

**Figure 2:**
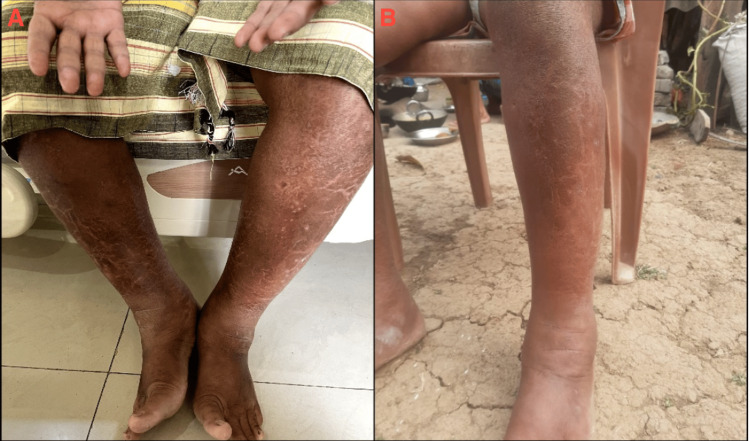
Edema of the lower extremities with skin changes at presentation (Left panel) and resolution after treatment (Right panel) A: Edema of the lower extremities with skin changes at presentation B: Resolution of the edema and skin changes after treatment

A 41-year-old male patient with no history of immunosuppression presented with a one-year history of progressive anasarca, hypopigmented skin lesions, and hair loss as shown in Figures 1, 2. He reported no recent travel but occasional consumption of untreated well water, raising suspicion for parasitic infection. Over the past six months, he had gained 12 kgs and experienced increasing fatigue, with significant periorbital swelling developing in the past month. He had no prior medical history, including diabetes mellitus, hypertension, or tuberculosis. On examination, his vital signs were stable, and findings included pallor, generalized edema affecting the face, abdomen, and scrotum, bilateral pitting pedal edema, diffuse scaling with hypopigmentation, and pruritus on the abdomen and lower limbs, suggesting a possible nutritional deficiency. 

The laboratory results (Table 1) revealed hypoproteinemia (total protein: 4.0 g/dL; reference range: 6.4-8.3 g/dL) and hypoalbuminemia (albumin: 1.6 g/dL; reference range: 3.5-5.0 g/dL).

**Table 1:** Summary of the laboratory parameters and radiological investigation RBC: Red blood cells; HBsAg: Hepatitis B surface antigen; HCV: Hepatitis C virus; HIV I & II: Human Immunodeficiency Virus types 1 and 2

Parameter	Result	Reference range
Hemoglobin (g/dL)	8.6	Male: 13.8–17.2, Female: 12.1–15.1
Total Leucocyte Count (TLC) (cells/mm³)	8,310	4,000–11,000
Platelet count (cells/mm³)	1,70,000	1,50,000–4,50,000
Serum sodium(Na⁺) mEq/l	141	135–145
Serum potassium(K⁺) mEq/l	3.7	3.5–5.0
Blood urea (mg/dL)	8.1	7–20
Serum creatinine (mg/dL)	0.6	0.6–1.3
Total bilirubin (mg/dL)	0.37	0.1–1.2
Direct bilirubin (mg/dL)	0.28	0.1–0.4
Alanine transaminase (ALT) (IU/L)	24	7–56
Aspartate transaminase (AST) (IU/L)	32	10–40
Alkaline phosphatase (ALP) (IU/L)	88	44–147
Gamma-glutamyl transferase (GGT) (IU/L)	143	9–48
Prothrombin time (PT) (in sec)	14	11–14 sec
International normalized ratio (INR)	1.22	0.8–1.2
Serum albumin (g/dL)	1.6	3.5–5.0
Serum calcium (mg/dL)	7.1	8.5–10.5
Peripheral smear	Normocytic normochromic anemia	Normocytic normochromic RBCs
Urine routine and microscopy	Pus cells – 0, RBC – Nil, Urine – Trace	
Stool routine and microscopy	Mucus – absent, Blood – absent, RBCs – nil, Pus cells – 0-1, Protozoa – present (*Giardia lamblia *cyst)	
24-hour urinary protein (g/day)	0.15	<0.15
HBsAg/HCV/HIV I & II	Negative	Negative
Serum Vitamin B12 (pg/mL)	<159	200–835
Anti-tTG (anti-tissue transglutaminase antibodies) IgA	Negative	Negative
C-reactive protein (CRP) (mg/L)	5.3	CRP: <10 mg/L
Erythrocyte sedimentation rate (ESR) (mm/hr)	10	ESR: <20 mm/hr (male), <30 mm/hr (female)
Upper GI Endoscopy	Normal	
Colonoscopy	Normal	
CT Enterography	Diffuse parietal wall oedema with fat stranding	
Biopsy of small intestine	Normal (no villous atrophy)	

Both colonoscopy and upper gastrointestinal endoscopy were unremarkable, while the abdominal ultrasound showed mild to moderate ascites. CT enterography revealed diffuse parietal wall edema with fat stranding and a left-sided pleural effusion. A duodenal biopsy showed no villous atrophy or other abnormalities, and urinalysis confirmed normal protein excretion. Liver stiffness and hepatic steatosis were assessed using vibration-controlled transient elastography (VCTE) ultrasound with FibroScan^®️^ (Model 502, Echosens, Paris, France) and showed a normal parenchymal echo texture (score: 5.3 kPa).

Notably, the stool examination revealed pathognomonic *Giardia lamblia *cysts, and a stool immunochromatography test confirmed a positive Giardia antigen. The patient was diagnosed with PLE secondary to giardiasis and was treated with a single oral dose of tinidazole 2 g along with intravenous albumin to address hypoproteinemia and the generalised edema.

At the two-month follow-up, the patient showed significant improvement, with resolution of the edema, weight loss, an increase in albumin levels from 1.6 g/dL to 2.7 g/dL, negative stool cultures, and no further gastrointestinal symptoms. This case highlights the importance of considering infectious causes such as giardiasis in unexplained PLE.

**Figure 3:**
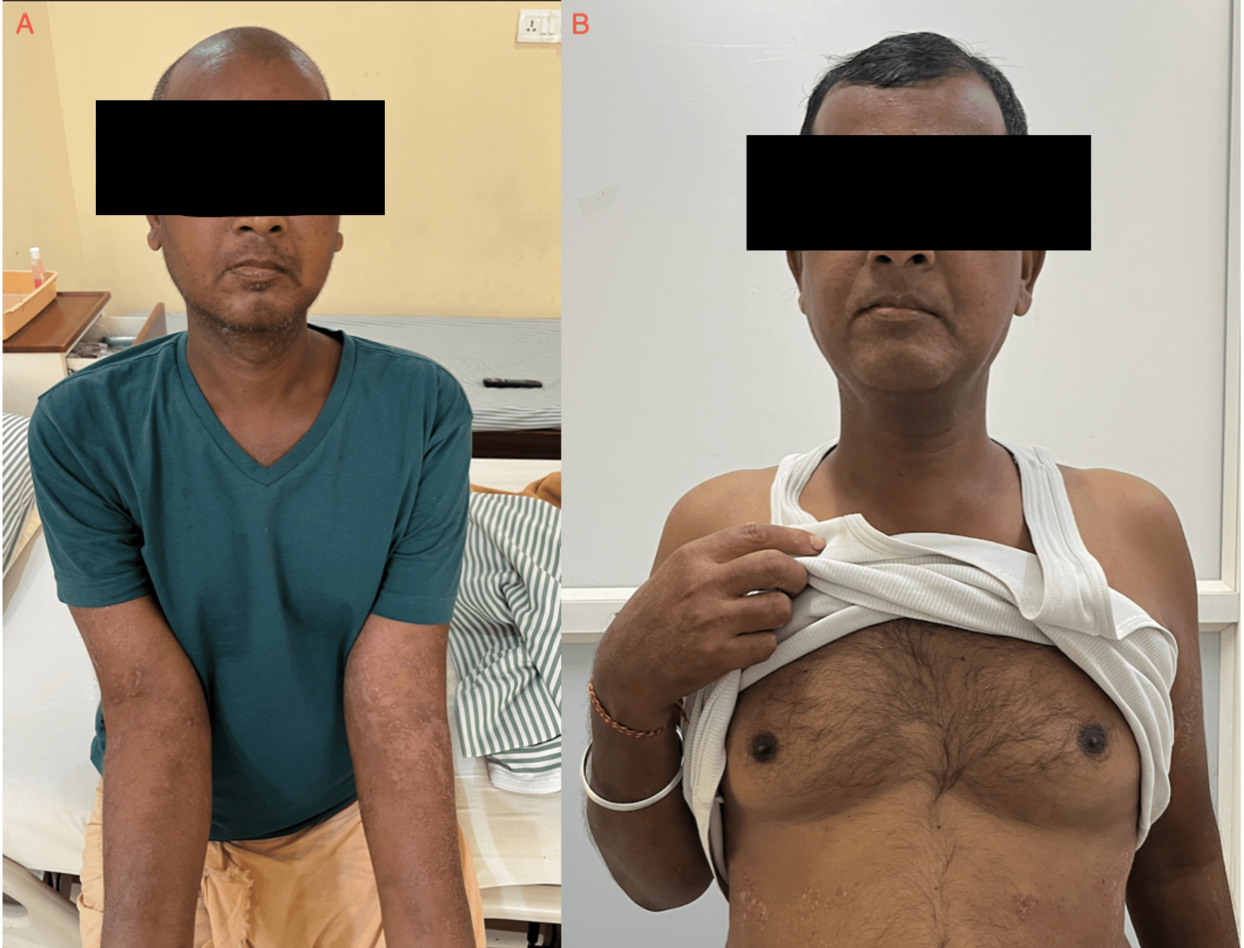
Facial features and hair condition A: Facial features at the time of presentation B: Improvement in the patient's general appearance with hair growth after one year

The patient showed up for follow up after one year (Figure 3). He was doing well and his total protein and albumin were within normal range. The skin lesions had disappeared and the generalized body swelling had abated completely.

## Discussion

*Giardia lamblia* is a flagellated intestinal protozoan transmitted via the fecal-oral route. Infection occurs primarily in the proximal small intestine, particularly the duodenum, where motile trophozoites attach to enterocytes [8]. Unlike invasive pathogens, it disrupts tight junctions and induces villous blunting, increasing intestinal permeability.

This case demonstrates how severe mucosal injury can lead to PLE, akin to rare reports of *Cryptosporidium*-induced enteropathy. The parasite’s trophozoites attach to the intestinal lining, particularly in the upper small intestine, causing structural and functional damage. This may range from no visible changes to severe villous atrophy. The intestinal lining may show deeper crypts, shorter microvilli, and disrupted tight junctions, leading to increased permeability. This damage can reduce key digestive enzymes like lactase, contributing to malabsorption. Even after successful treatment, these enzyme deficiencies may take time to recover [9]. Disruption of the gut barrier and microbiota balance may also play a role in the symptoms [10].

There is also an increasing recognition of the role of host factors in determining the outcome of infection. Studies have identified some genetic factors of the host that influence susceptibility to chronic infection. For instance, variations in the immune response genes, such as those regulating the production of interleukins (IL-4, IL-10), may predispose individuals to persistent giardiasis. Chronic infections, particularly in immunocompromised individuals, can lead to malabsorption, malnutrition, and other long-term health complications [4]. The clinical spectrum of giardiasis ranges from asymptomatic carriage to severe diarrhea and malabsorption. Acute infection typically presents with watery diarrhea, malaise, foul-smelling and fatty stool, abdominal cramps, bloating, nausea, and weight loss, which generally last for one to four weeks [11]. However, for some, the infection becomes chronic, leading to persistent gastrointestinal symptoms, weight loss, and malnutrition. Chronic giardiasis may follow acute infection and may sometimes without an antecedent acute illness [12].

PLE is a rare complication of giardiasis and is primarily reported in infants. The severe infection damages the enteric mucosa, leading to an exudative protein loss [1]. A duodenal biopsy may reveal a varied appearance of the mucosa, ranging from normal to severe villous atrophy with mononuclear cell infiltrates, similar to celiac disease [13]. Other pathogens inducing exudative enteropathy in immunocompromised adults and children include *Cryptosporidium*, Gram-negative organisms, and *Entamoeba histolytica* [14].

In this case, the patient presented with anasarca, skin changes, and weight gain, symptoms which are not typically associated with giardiasis. Prompt recognition of these symptoms attributable to hypoproteinemia, coupled with appropriate diagnostic testing, facilitated timely treatment initiation and prevented potential complications from prolonged infection.

The diagnosis of giardiasis typically involves the detection of *Giardia* cysts or trophozoites in the stool samples. Traditional diagnostic methods include light microscopy and stool antigen detection assays, but these have limitations in terms of sensitivity, selection of direct or concentration methods, the number of fecal samples examined, and the expertise of trained technicians. The intermittent nature of cyst excretion must also be considered [15]. Molecular diagnosis of *Giardia* typically relies on amplifying target genes (using techniques such as conventional polymerase chain reaction (PCR), nested PCR, or real-time PCR. More specialized methods-like PCR-Restriction Fragment Length Polymorphism (RFLP), Random Amplified Polymorphic DNA (RAPD)-PCR, or DNA hybridization can further differentiate *Giardia *assemblages and aid in epidemiological tracking [16]. Serological testing including enzyme-linked immunosorbent assays (ELISA), immunofluorescence, loop-mediated isothermal amplification (LAMP) have also been explored as diagnostic tools, with recent advancements identifying specific biomarkers for active infection [15].

The mainstay of treatment for giardiasis-related PLE is the administration of anti-parasitic medications. Tinidazole and metronidazole are the most commonly used drugs, with tinidazole often preferred due to its single-dose regimen and better patient compliance [17]. In this instance, the patient received a single oral dose of tinidazole 2 g alongside intravenous albumin, likely contributing to the faster resolution of edema and symptoms.

A meta-analysis of 60 randomized trials involving both adults and children with giardiasis demonstrated higher parasitological cure rates with tinidazole compared to metronidazole [17]. In cases where oral medications are intolerable, intravenous metronidazole may serve as an alternative. However, resistance to metronidazole has been reported in certain regions [18]. Newer treatments are being explored, including the use of combination therapies and repurposed drugs. Studies have evaluated the effectiveness of drugs like albendazole and secnidazole for giardiasis, with promising results in terms of efficacy and tolerability [19]. In addition, adjunctive therapies such as probiotics are being investigated to restore gut microbiota balance in patients with chronic or recurrent giardiasis [20].

Prevention of giardiasis primarily focuses on improving water quality and hygiene practices. *Giardia* cysts are eliminated by boiling tap water for at least one minute. Public health campaigns aimed at improving hand hygiene and raising awareness about the risks of drinking untreated water are critical, especially in endemic areas [20]. For hikers and campers, water purification tools include water filtration, iodination, and chlorination, where iodine-based treatments are more effective [22]. In recent years, there has been a push towards developing vaccines for giardiasis. While a vaccine is not yet available, ongoing research into the immunology of *Giardia* infection is gradually moving closer to identifying potential vaccine candidates. Studies have highlighted the role of surface antigens such as *Giardia *cyst wall proteins and trophozoite adhesive proteins as possible targets for vaccine development [23].

## Conclusions

Early recognition of giardiasis as a potential cause of PLE is essential for effective treatment. Given that giardiasis-related PLE can present with nonspecific gastrointestinal and systemic symptoms, it should be considered in the differential diagnosis, particularly when more common causes of protein loss, such as inflammatory bowel disease or liver cirrhosis have been ruled out. *Giardia* should be ruled out in unexplained PLE via stool antigen/PCR, even without diarrhea. Post-treatment albumin monitoring is advised, as delayed recovery may suggest concurrent malabsorption or secondary infections. With timely intervention, most patients with giardiasis-induced PLE achieve full recovery, highlighting the importance of recognising this uncommon manifestation of a common parasitic infection. 
